# Distal retinal ganglion cell axon transport loss and activation of p38 MAPK stress pathway following VEGF-A antagonism

**DOI:** 10.1038/cddis.2016.110

**Published:** 2016-05-05

**Authors:** R Foxton, A Osborne, K R Martin, Y-S Ng, D T Shima

**Affiliations:** 1 Department of Ocular Biology and Therapeutics, University College London, Institute of Ophthalmology, London, UK; 2 John van Geest Centre for Brain Repair, University of Cambridge, Cambridge, UK; 3 Wellcome Trust Medical Research Council Cambridge Stem Cell Institute, Cambridge, UK; 4 Cambridge NIHR Biomedical Research Centre, Cambridge, UK; 5 Eye Department, Addenbrooke's Hospital, Cambridge, UK; 6 The Schepens Eye Research Institute and Massachusetts Eye and Ear, Harvard Medical School, Boston, MA, USA

## Abstract

There is increasing evidence that VEGF-A antagonists may be detrimental to neuronal health following ocular administration. Here we investigated firstly the effects of VEGF-A neutralization on retinal neuronal survival in the Ins2^Akita^ diabetic and JR5558 spontaneous choroidal neovascularization (CNV) mice, and then looked at potential mechanisms contributing to cell death. We detected elevated apoptosis in the ganglion cell layer in both these models following VEGF-A antagonism, indicating that even when vascular pathologies respond to treatment, neurons are still vulnerable to reduced VEGF-A levels. We observed that retinal ganglion cells (RGCs) seemed to be the cells most susceptible to VEGF-A antagonism, so we looked at anterograde transport in these cells, due to their long axons requiring optimal protein and organelle trafficking. Using cholera toxin B-subunit tracer studies, we found a distal reduction in transport in the superior colliculus following VEGF-A neutralization, which occurred prior to net RGC loss. This phenomenon of distal transport loss has been described as a feature of early pathological changes in glaucoma, Alzheimer's and Parkinson's disease models. Furthermore, we observed increased phosphorylation of p38 MAPK and downstream Hsp27 stress pathway signaling in the retinas from these experiments, potentially providing a mechanistic explanation for our findings. These experiments further highlight the possible risks of using VEGF-A antagonists to treat ocular neovascular disease, and suggest that VEGF-A may contribute to the maintenance and function of axonal transport in neurons of the retina.

Vascular endothelial growth factor (VEGF-A) antagonists were originally developed to treat cancer, before being used for ocular application in wet age-related macular degeneration (wet AMD).^1^ Their use in the eye has since expanded, and they are now approved for diabetic macular edema, retinal vein occlusion, plus given off-label for conditions such as neovascular glaucoma. These drugs' mechanism of action is to neutralize pathological increases in VEGF-A, thus removing a potent angiogenic stimulus and source of vascular hyperpermeability.

VEGF-A was initially named following discovery of its potent effects on endothelium. However, contrary to its 'vascular'-specific title, VEGF-A acts on multiple tissues, including in the nervous system. VEGF-A is neurogenic and neuroprotective in a variety of different cell types, *in vitro* and *in vivo*.^2, 3, 4, 5^ These findings raised the possibility that blocking VEGF-A when treating neovascular disease could adversely deprive neurons of an essential survival factor.

We initially investigated this in models of ischemia–reperfusion injury,^6^ then ocular hypertension (OHT), to parallel a disease scenario where VEGF-A antagonists are used in the clinic, namely for neovascular glaucoma, or as an adjunct to trabeculectomy surgery.^7^ We found in both models that exogenously injected VEGF-A was protective, but also VEGF-A-neutralization exacerbated retinal damage. This indicates tissue sources of VEGF-A are critical to neuronal survival^6, 7^ and part of the tissue's response to injury. Furthermore, using cultured purified primary retinal ganglion cells (RGCs), we discovered that VEGF-A directly protected retinal neurons against H_2_O_2_-induced caspase-3-mediated apoptosis via VEGFR-2 signaling and the Pi3-kinase/Akt axis.^7^

These data lead us to consider the impact of anti-VEGF-A therapy in diabetes and wet AMD, the biggest areas of treatment in terms of patient numbers, and also investigate possible causes of neuron death. In this report we examined cell death following anti-VEGF-A treatment in Ins2^Akita^ diabetic^8^ and JR5558 spontaneous choroidal neovascularization (CNV) mice.^9^ We established that VEGF-A antagonists also accelerate neuronal apoptosis in these models, findings that may have implications for treating ocular neovascular disease with anti-VEGF drugs.

We next sought to find potential mechanisms contributing to neuron loss. We examined the effect of VEGF-A neutralization on synapses in the inner and outer plexiform layers (INL and ONL, respectively) and also axon transport along the visual pathway. RGCs have long axons that project uninterrupted from the retina to the superior colliculus (SC) in rodents, and as such require efficient transport to function well. We used cholera toxin B subunit (CTB) to quantify anterograde transport in the retina, optic nerve and SC of rats treated with a VEGF-A neutralizing agent. Furthermore, we investigated activation of p38 mitogen-activated kinase (p38 MAPK) and downstream heat-shock protein 27 (Hsp27) pathways in VEGF-A antagonist treated retinas, which have been reported to contribute to anterograde transport loss in glaucoma models.^10^ Our findings suggest that VEGF-A antagonists cause a distal reduction of RGC transport and activation of p38 MAPK, which may mediate these effects. We believe that our experiments are important for providing further insight into the role of VEGF-A in retinal neuronal homeostasis.

## Results

### VEGF-A neutralization exacerbates cell death in diabetic and spontaneous CNV models

**Figure 1:**
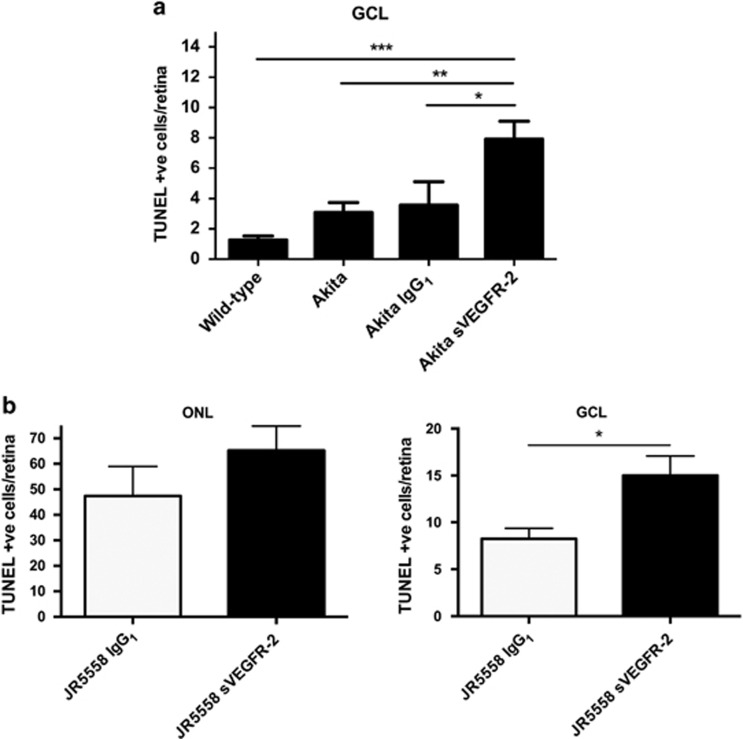
VEGF-A antagonism increases neuronal death in Ins2^Akita^ diabetic and JR5558 spontaneous CNV models. (**a**) VEGF-A neutralization, via intravitreal injection of 4 pmol sVEGFR-2, significantly elevated RGC death in the Ins2^Akita^ mouse model, above IgG_1_ and uninjected controls, plus wild-type. *N*=7–15. (**b**) sVEGFR-2 administration in the JR5558 eye had no significant effect on TUNEL staining in the ONL (left graph), but in the GCL apoptosis was increased 1.8-fold above IgG_1_ control (right graph). *N*=12–14. *P<0.05, **P<0.01, ***P<0.001. Data are given as means±S.E.M.

Previously we showed VEGF-A antagonism accelerates neuronal death in a disease model of glaucoma. Here we extend these findings to models of diabetic retinopathy, as well as spontaneous CNV, akin to wet AMD, which unlike the glaucoma model have VEGF-driven pathology.^9, 11^ We used Ins2^Akita^ diabetic mice, which have elevated levels of apoptotic nuclei in the ganglion cell layer (GCL) (Supplementary Figure S1A; *P*<0.01). After two weekly intravitreal injections, a significant 2.2-fold increase in RGC apoptosis from 3.6±1.5 to 7.9±1.2 cells/retina in the GCL above IgG_1_ control was observed following soluble VEGFR-2/Fc chimera (sVEGFR-2) treatment (Figure 1a; *P*<0.01).

Following this, we examined whether these findings applied to spontaneous CNV mice. JR5558 have abundant photoreceptor apoptosis surrounding neovascularization,^9^ and higher levels of RGC death compared with wild-type (Supplementary Figure S1B; *P*<0.01). Treatment with sVEGFR-2 by intravitreal injection significantly reduced both CNV area and number by approximately 50% compared with IgG_1_ control (Supplementary Figures S1D and E; *P*<0.01). Neuronal death in the JR5558 was unchanged in the outer nuclear layer (ONL) between-treatment groups (Figure 1b); however, in the GCL apoptotic nuclei rose significantly 1.8-fold (Figure 1b; *P*<0.05) following sVEGFR-2 (8.3±1.1 to 15.0±2.1 cells/retina). Interestingly, increases in cell death following VEGF-A antagonist treatment were also observed in wild-type animals serving as controls for both Ins2^Akita^ and JR5558, which significantly rose from 1.3±0.3 and 2.0±0.8 in untreated and IgG treated retinas, respectively, to 8.0±2.0 cells/retina in the GCL following sVEGFR-2 (Supplementary Figure S1C; *P*<0.01). This indicates RGC loss may occur after VEGF-A blockade independently of the model used.

### Retinal synapse architecture is not affected by VEGF-A neutralization

Our data show that VEGF-A antagonists also elevate neuronal death in models of diabetes and wet AMD; however, the cause of neuron loss in these models has not yet been examined. Is retinal neuron death the consequence of removing an essential survival factor, or does VEGF-A influence the normal physiology of these cells? We investigated two properties of retinal neurons: synaptic architecture and axon transport, which are critical to the normal functioning of the cells.

VEGF-A has been reported to regulate synapses through enhancing synaptic transmission *in vitro*^12^ and modulating synaptic plasticity in development independently of neuro- and angiogenesis in the adult mouse hippocampus.^13^ With this in mind, we investigated if VEGF-A blockade may alter the synaptic architecture of retinal inner and outer plexiform layers (IPL and OPL, respectively). These synaptic connections can be deregulated in ocular disease models,^14^ so we used normal C57Bl/6J mice to avoid this confounding influence. Quantification of presynaptic synpatophysin, postsynaptic PSD-95, then colocalization of the two markers as synaptic 'puncta' (Supplementary Figure S2A), following intravitreal injection of sVEGFR-2 or IgG_1_, was carried out as previously described.^15^ No significant difference between treatment groups was observed in either IPL (Supplementary Figure S2B) or OPL (Supplementary Figure S2C), at central, medial or peripheral sites of the retina (left, middle and right panels, respectively). These data indicate that acute VEGF-A antagonism does not affect gross synaptic architecture in the adult mouse retina.

### Anterograde axonal transport is disrupted distally in the visual pathway

We have consistently observed increased cell death in RGCs in our studies. These cells have lengthy axons that project from the retina, primarily to the SC in rodents, and require optimal axonal transport to function effectively. Using CTB labeling, we investigated whether VEGF-A antagonism disrupts anterograde transport in RGCs. We used normal female Brown-Norway rats to avoid complicated interactions of pathology on axon transport, which are compromised in many disease models.^16, 17^ We injected rat eyes intravitreally with either IgG_1_ or sVEGFR-2, then 7 days later administered CTB via the same route and harvested the tissue at 4, 8, 24 and 48 h post-CTB.

**Figure 2:**
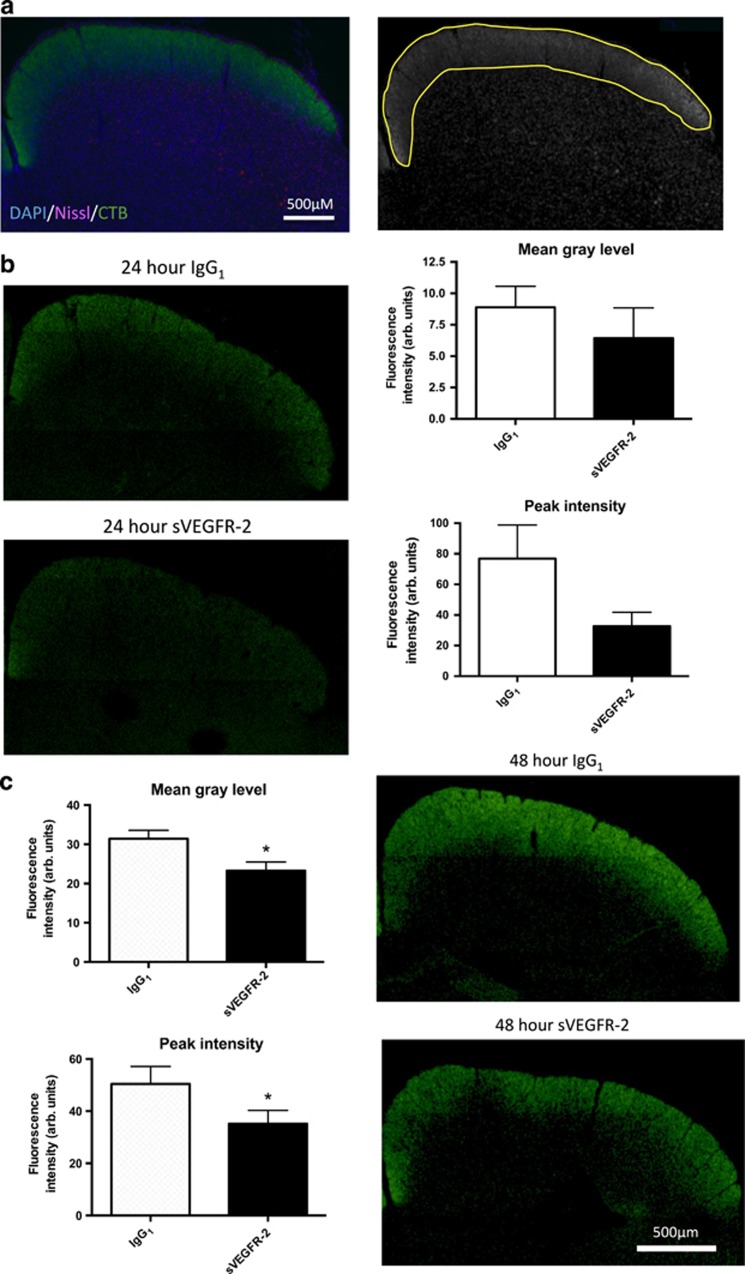
VEGF-A neutralization reduces cholera toxin B (CTB) labeling in the superior colliculus. Brown-Norway rats were injected bilaterally IVT with 20 pmol of either IgG_1_ or sVEGFR-2, and 7 days later received IVT 2 *μ*l of 0.75% CTB. Brains were harvested and frozen coronal sections of the SC prepared. (**a**) Quantification methodology. Frozen sections were stained with Nissl (magenta) and DAPI (blue) (left panel) to allow unbiased selection of SC regions (right panel), even when CTB signal (green) was weak. Left and right SC of each brain slice were analyzed independently. (**b**) At 24 h post-CTB injection, no significant differences between treatment groups were observed in the mean gray level (8.8±1.7 *versus* 6.4±2.4; IgG_1_ *versus* sVEGFR-2, respectively; top graph), or peak fluorescence intensity (76.8±22.0 *versus* 32.7±9.1; IgG_1_ *versus* sVEGFR-2, respectively; bottom graph)(both arbitrary units). Left panels show representative images of CTB labeling with these treatments. (**c**) At 48 h post-CTB significant reductions were seen in both mean gray level (31.4±2.2 *versus* 23.3±2.2; IgG_1_ *versus* sVEGFR-2, respectively; top graph) and peak intensity (50.4±2.8 *versus* 35.2±5.1; IgG_1_ *versus* sVEGFR-2, respectively; bottom graph) in the sVEGFR-2 treatment group compared with IgG_1_. Right panels; representative images of CTB labeling 48 h post-injection of IgG_1_ (top panel) or sVEGFR-2 (bottom panel) treatment groups. *P<0.05, *N*=6. Data are given as means±S.E.M. Original magnification= × 4

First we quantified accumulation of CTB in RGC axon termini in the SC (Figure 2a), shown as overall fluorescence intensity (mean gray level) and maximum fluorescence (peak intensity) in the graphs. At 4 and 8 h after injection, CTB was not present in the SC (data not shown), presumably as it had not yet travelled the full length of RGCs. At 24 h post-injection clear CTB labeling was present (Figure 2b, left panels), and although there was a trend toward a decrease in peak intensity of CTB staining following sVEGFR-2 (Figure 2b, bottom right panel; *P*=0.09), there was no statistically significant difference between the groups in the overall mean gray fluorescence level (Figure 2b, top right panel). At 48 h after CTB, build up of the tracer had increased further (Figure 2c, right panels); however, this time there was a significant reduction in CTB accumulation in sVEGFR-2 treated eyes. Both mean gray level and peak intensity (Figure 2c, left panels) were reduced in comparison with IgG_1_, indicating anti-VEGF-A treatment may attenuate distal axon transport in RGCs. The temporal profile of CTB accumulation in IgG_1_ and sVEGFR-2 treated retinas indicates that fast axonal transport may be affected,^18^ as differences were observed at just 48 h post-CTB injection.

### Distal anterograde transport deficit is not caused by reduced uptake of CTB into RGCs

**Figure 3:**
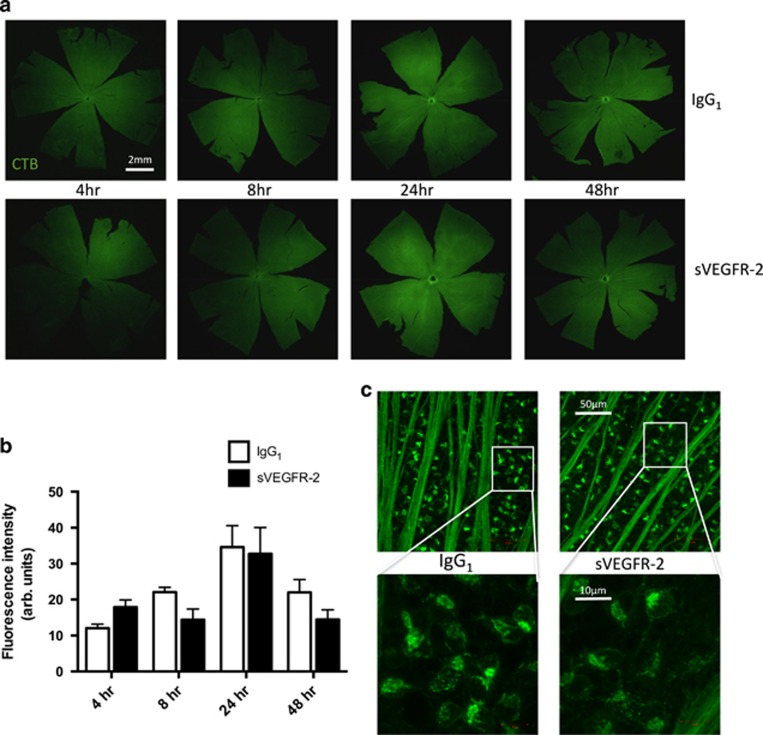
Anti-VEGF-A treatment does not affect CTB uptake into the retina. (**a**) Rat eyes were bilaterally injected IVT with 20 pmol IgG_1_ or sVEGFR-2, then 7 days later with 2 *μ*l of 0.75% CTB. The tissue was dissected, flat mounted and confocal tile scans made of entire retinas harvested at 4 (left panels), 8 (middle left panels), 24 (middle right panels) and 48 h (right panels) post-CTB injection. Original magnification= × 4. (**b**) Quantification of mean gray levels indicated an increase in fluorescence intensity of CTB in the retina from 4 to 24 h post-injection; however comparison of IgG_1_ and sVEGFR-2 groups revealed no significant differences between treatments at any of the time points. (**c**) Higher magnification images of RGCs showed no variation in intracellular accumulation of CTB using × 10 (upper panels) or × 40 (lower panels) magnification at 48 h post-CTB injection, with IgG_1_ (left panels) or sVEGFR-2 (right panels) treatment. White boxes on upper panels indicate regions that were imaged using × 40 objective for the lower panels. *N*=6–10. Data are given as means±S.E.M.

A number of neurodegenerative disease models are characterized by axon transport deficits, often manifesting as early signs of pathology. These frequently first appear in the distal portion of neurons, then progress to more proximal regions.^16, 19, 20^ We looked at the dynamics of CTB transport elsewhere in the visual pathway to see if the transport loss was observed in more proximal regions; firstly in the retina, then optic nerve. CTB is taken into RGCs via GM1 ganglioside receptor binding,^21^ and we firstly quantified tracer fluorescence in the retina as a readout of uptake. We generated images of the whole retina using confocal microscopy, to quantify CTB in eyes from these studies, at 4, 8, 24 and 48 h post-injection (Figure 3a). Fluorescence intensity appeared to increase over time, up to 24 h after delivery of tracer, then decline at 48 h. We found no significant difference in CTB uptake between treatment groups at any of the time points (Figure 3b). High-magnification images of retinas at 48 h post-injection showed a similar distribution of CTB in RGCs of IgG_1_ *versus* sVEGFR-2 treated eyes (Figure 3c). These data indicate that uptake of CTB into the retina is not affected by VEGF-A antagonism.

### Levels of CTB in the optic nerve are unaffected by VEGF-A neutralization

**Figure 4:**
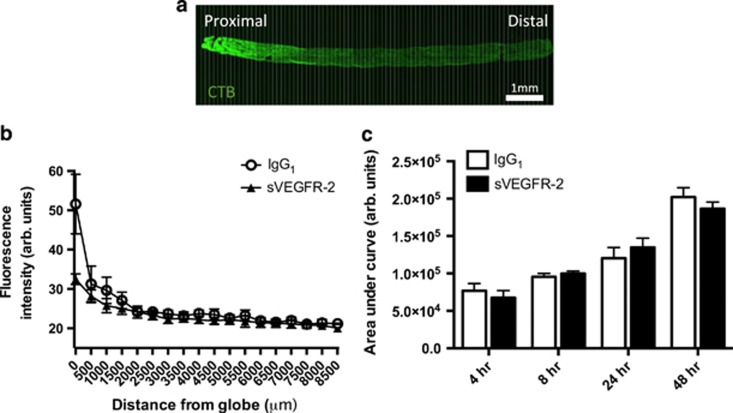
CTB transport in the proximal portion of the optic nerve is not affected by VEGF-A neutralization. (**a**) Tile scan confocal images of longitudinal sections of the entire optic nerve, between the posterior globe and optic chiasm, were used to quantify CTB anterograde transport along the optic nerve (top panel). Measurements of fluorescence intensity were taken at 100 *μ*m intervals (bottom panel**;** white vertical lines representing 100 *μ*m), plotted over the length of the tissue (**b**); data from 48 h post-CTB injection time point, and used to calculate the area under the curve (**c**). AUC fluorescence intensity increases with time post-CTB injection; however, no significant differences were observed at any of the time points between IgG_1_ and sVEGFR-2-treatment groups. For presentation purposes, only data points every 500 *μ*m are shown in (**b**); however, AUC was calculated from 100 *μ*m intervals along the optic nerve. *N*=3–10 optic nerves. Data are given as means±S.E.M. Original magnification= × 4

CTB staining in the optic nerve also did not appear to be affected by sVEGFR-2 IVT injection. Area under the curve analysis of fluorescence intensity from longitudinal optic nerve sections (Figures 4a and b) showed that CTB uptake increased over time post-CTB injection in the optic nerve, from 4 to 48 h. Pre-treatment with the VEGF-A antagonist had no significant impact on this accumulation, either at 4, 8, 24 or 48 h (Figure 4c).

Taken together, the data from SC, retina and optic nerve indicate that anti-VEGF-A induces a distal loss of CTB labeling along RGC projections, which after a single acute dose of sVEGFR-2 does not manifest in proximal transport disruption.

### Axonal transport changes precede cell loss

**Figure 5:**
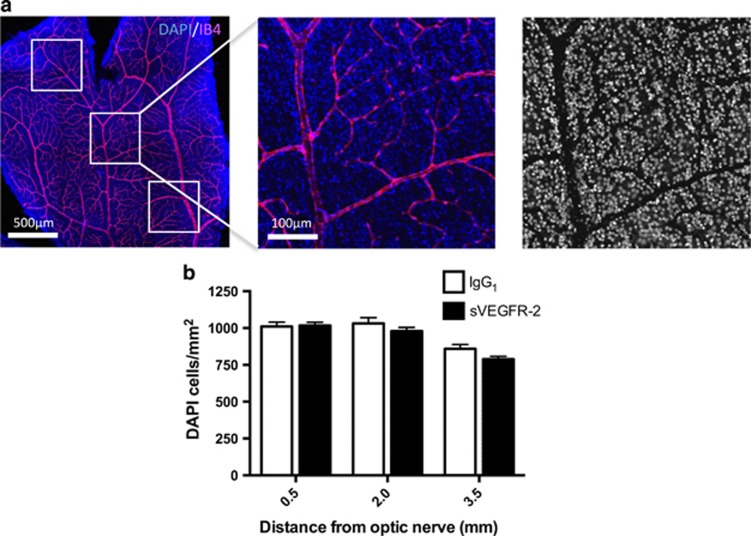
Reductions in CTB transport precede cell body loss. (**a**) Retinas from 48- h post-CTB injection transport experiments were stained with DAPI (blue) and IB4 (magenta) (left panel). Tile scan images of each of the four petals were taken (original magnification= × 10), 0.5mm^2^ areas were selected at 0.5, 2.0 and 3.5mm from the optic nerve (middle panel), and then processed to remove the vascular area from each picture (right panel). DAPI-stained nuclei were counted using ImageJ software. (**b**) A comparison of DAPI nuclei per mm^2^ revealed no significant differences between IgG_1_ or sVEGFR-2 treatments at 0.5, 2.0 or 3.5mm from the optic nerve. These data indicate that at 1-week post-sVEGFR-2 injection, transport deficits may precede significant RGC loss. *N*=5–6 retinas. Data are given as means±S.E.M.

Next, we wanted to determine whether changes in axon transport were early signs of injury, or if they accompanied significant neuron loss in the GCL. Tissue was stained with DAPI (4',6-diamidino-2-phenylindole), and high-resolution images were generated to quantify RGCs in central, medial or peripheral locations in whole-mounted retinas (Figure 5a). Isolectin-B4 counterstaining excluded retinal vessels from cell counts. We observed no significant difference in neuron number at any of the separate regions of the retina (Figure 5b), suggesting that changes in anterograde transport occur early, prior to soma loss.

### Increased phosphorylation of p38 MAPK and Hsp27, but not Tau, accompany transport deficits

Finally, we looked for evidence of activation of signaling pathways that might contribute to distal anterograde transport loss following VEGF-A neutralization. P38 mitogen-activated protein kinases (p38 MAPK) are a class of MAPK responsive to stress signaling, and phosphorylation of these has a strong association with neurodegenerative disease.^22, 23^ In the retina, elevated phosphorylated p38 MAPK (p-p38 MAPK) and downstream targets, Hsp27 and Tau, are associated with RGC injury and loss of anterograde transport in rodent models of OHT.^10, 24^ With this in mind, we did immunostaining to localize phosphorylated p38 MAPK, Hsp27 and Tau on retinas from anterograde transport experiments.

**Figure 6:**
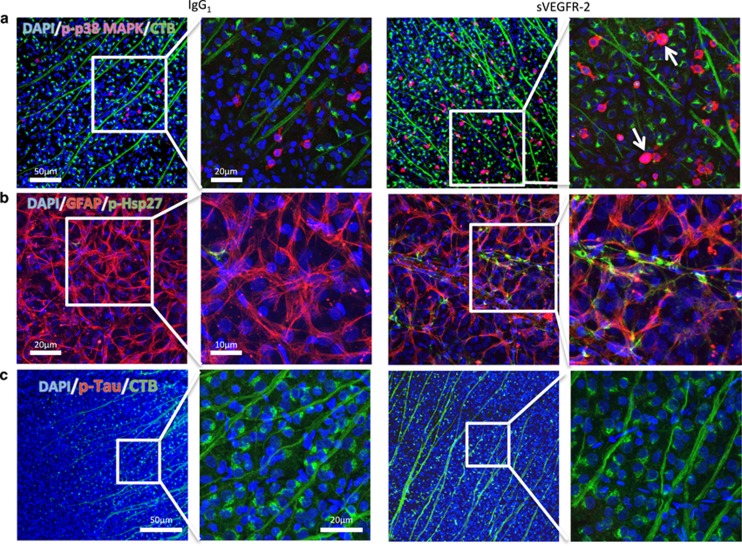
Changes in axon transport are accompanied by increased expression of phosphorylated p38 MAPK and downstream Hsp27, but not Tau. (**a**) Immunolocalization of phosphorylated p38 MAPK (p-p38 MAPK; magenta), in retinas from the 48-h post-CTB injection transport studies, revealed increased expression in eyes treated with sVEGFR-2 (right panels), in comparison with IgG_1_ controls (left panels). Original magnification= × 10; green=CTB, blue=DAPI. Higher magnification ( × 40) images (left middle and right) showing elevated p-p38 MAPK as well as nuclear localization in some cells (arrows) in sVEGFR-2 treated retinas. (**b**) Differences in expression of phosphorylated Hsp27 were also observed between IgG_1_ (left panels) and sVEGFR-2 (left panels) treatment. Phosphorylated Hsp27 (green) was strongly upregulated following sVEGFR-2 administration, and colocalized with GFAP (red),^41^ indicating astrocyte expression. Blue=DAPI. Left and right middle panels – original magnification= × 10; left middle and right panels – original magnification= × 40. (**c**) In contrast, no changes were detected in phosphorylated Tau levels between IgG_1_ (left panels) and sVEGFR-2 (right panels). Red=p-Tau, green=CTB, blue=DAPI. Original magnifications= × 10 (left and right middle) and × 40 (left middle and right)

We observed low background perinuclear p-p38 MAPK immunofluorescence in the retina after IgG_1_ administration (Figure 6a, left panels); however, in sVEGFR-2 treated tissue this was substantially increased, both in terms of level and number of cells expressing p-p38 MAPK (Figure 6a, right panels), with some cells exhibiting strong nuclear localization (arrows). Further examination of this staining revealed these cells to be mostly GFAP-positive, indicating astrocyte expression (Supplementary Figure S3A). However, a lesser number of cells were co-labeled with CTB, signifying RGCs also upregulated p-p38 MAPK (Supplementary Figure S3B).

Immunostaining for phosphorylated Hsp27 also revealed activation of this downstream target. In IgG_1_ treated eyes (Figure 6b, left panels), low level of Hsp27 phosphorylation was observed, but after sVEGFR-2 treatment intense staining was observed in cells sitting on top of RGCs (Figure 6b, right panels). GFAP colocalization revealed these cells to be astrocytes. In contrast, we were unable to see any change in hyperphosphorylated Tau protein, another target of p38 MAPK, perhaps indicating a more specific downstream activation of Hsp27. In the optic nerve and SC (data not shown) we found no differences in p-p38 MAPK, p-Hsp27 or p-Tau levels in between-treatment groups. These data show that p38 MAPK signaling pathways are activated in the retina in response to VEGF-A antagonism.

## Discussion

We provide here additional evidence that VEGF-A is a critical survival factor for neurons, and that neutralization can exacerbate cell death. Previous work by ourselves and others demonstrated this in the neural retina following VEGF-A depletion via local or systemic injection of neutralizing agents,^6, 7, 25^ which did not affect normal adult vasculature but enhanced neuronal apoptosis in the inner and outer retina. We show here in Ins2^Akita^ type I diabetic and JR5558 spontaneous CNV mouse models that even when the disease phenotype responds to anti-VEGF-A treatment (as the neovascular lesions in the CNV mouse did) neuronal cell death is exacerbated not diminished.

VEGF-A levels are increased in the eye in both the diabetic and CNV models,^9, 11^ in line with patient data for both diabetic retinopathy^26^ and wet AMD.^1^ Pathologically elevated VEGF-A levels are a key feature of ocular neovascular disease, driving angiogenesis and vessel leakage. However, one possibility may be that in early stages VEGF-A is produced to protect neurons from degeneration. In a rat model of IOP elevation, diabetes was observed to be neuroprotective against acute RGC loss.^27^ Additionally epidemiological studies have proposed that the early stages of diabetes may be neuroprotective against glaucoma.^28, 29^ It is not yet clear if VEGF-A is linked to this protection; however, our evidence in disease models suggests that removing tissue sources of this molecule may be deleterious.

Does the increase in retinal neuron apoptosis with anti-VEGF-A simply reflect loss of a survival factor? The retina has a high metabolic demand,^30^ so that even under normal conditions cells may be greatly exposed to reactive oxygen and nitrogen species. Loss of VEGF-A, and downstream Pi3-kinase/Akt signaling, may leave cells vulnerable to insult. However, we also explored whether VEGF-A could have a more integral role in the normal physiology of the cells, by looking at two aspects of retinal neuron function: synapse architecture and anterograde axon transport. VEGF-A is reported to influence synaptic connectivity,^12, 13^ but after delivery of VEGF-A antagonist to control mice retinas we found no alteration in synaptic puncta number in IPL or OPL. This may be because VEGF-A is more critical in development, and perhaps is not essential for regulation of adult tissue.

Although synapse number did not alter, data from our anterograde transport studies indicate that RGCs lose some ability to transport molecules along the visual pathway following VEGF-A neutralization, an effect preceding reduction in neuron numbers. We observed a significant decrease in CTB-labeling in the SC, the distal projection site of RGCs, 48 h after CTB injection. In contrast, CTB uptake into the retina and along the proximal portion of the optic nerve prior to the optic chiasm did not diminish. Distal transport loss has been well described as an early sign of neuropathy in many disease models, involving progressive distal to proximal loss of function along axonal projections, followed by eventual degeneration of the soma. In the DBA/2J model of glaucoma, anterograde transport impairment is first observed in the SC, followed by the optic nerve and eventually the retina as the animals age and pathology worsens.^16^ Distal injury is also one of the earliest hallmarks of neuronal degeneration in models of Alzheimer's and Parkinson's disease, as well as amyotrophic lateral sclerosis.^19, 20, 31^

In this study, like others investigating anterograde transport along RGCs,^10, 16^ the site of transport reduction and axonal injury was not pinpointed. We did however observe alterations in retinal signaling following VEGF-A antagonist delivery that may provide some mechanistic understanding. Activation, through phosphorylation, of p38 MAPK, and downstream Hsp27 are associated with stress signaling, and contribute to axon transport deficits and pathology of both mouse and rat models of glaucoma.^32, 33^ Conversely, inhibition of p38 activation can prevent these transport losses.^10^ Following VEGF-A neutralization, we found strong expression of both phosphorylated p38 MAPK and Hsp27 throughout the inner retina. Expression was not limited to RGCs however, but found largely in astrocytes, so we are unable to specify how much of the axon transport deficits are the direct result p38 MAPK and Hsp27 activation in RGCs, or via paracrine signaling through glial cells. Interestingly, we also saw no evidence of Tau hyperphosphorylation, another downstream target of p38, which has a strong association with neurodegenerative processes and disease.^34, 35^

The contribution of these signaling pathways, and a more precise role of VEGF-A in axon transport, should be further evaluated. Currently there are a lack of well-established *in vitro* or *ex vivo* assays to allow monitoring of gross movement of molecules anterogradely in neurons, making it difficult to fully assess the contribution of VEGF-A. However, understanding these pathways may be significant, because even transient losses of anterograde transport or survival signaling could leave neurons vulnerable. Currently we are not aware of other studies investigating the role of VEGF-A in axon transport; however, there may be indirect evidence. An increase in sensory neuropathies has been reported in cancer patients receiving VEGF-A antagonists in combination with chemotherapy,^36, 37^ which is not attributed solely to chemotherapeutics.^38^ Nociceptors, like RGCs have long axons, and require efficient movement of proteins and organelles to function. It is possible VEGF antagonists interrupt transport along these sensory neurons, contributing to painful neuropathies.

Is there reason to be concerned about neuronal side effects of VEGF antagonists in ocular neovascular disease in humans? Typically clinical trials for diabetic macular edema and wet AMD do not include assessment of neuronal survival or function, so it is difficult to directly compare our results with clinical data. However, some studies have emerged looking at aspects of retinal health following anti-VEGF-A treatment. Notably, the Comparison of Age-Related Macular Degeneration Treatments Trials (CATT) monitored 1024 patients for signs of geographic atrophy (GA), who did not display this upon enrollment. After 2 years of bevicizumab or ranibizumab treatment, 18.3% of patients developed GA, leading the authors to conclude that anti-VEGF-A treatment may have a role in the development of GA.^39^ Additionally, the SEVEN-UP study, assessing 7–8 year outcomes in 65 AMD patients receiving ranibizumab treatment, found a mean decline in letter score of 8.6 letters, and in 37% of patients, visual acuity of 20/200 or worse.^40^ As yet, there are no similar studies for diabetic macula edema; however, given that diabetic patients begin treatment at a younger age, and therefore receive therapy more chronically, longitudinal studies could be initiated to monitor the health of these patient's eyes.

Currently VEGF-A antagonists still represent the best pharmacological treatment for wet AMD, diabetic macular edema and retinal vein occlusion, with the benefits very clearly outweighing the risks, at least in the short term. However, future drug discovery efforts for ocular neovascular disease should include extensive preclinical assessment of neuronal function and/or potential damage before entering into humans. Perhaps neuroprotective agents should be considered more widely for clinical use, in combination with agents that act on vascular targets, because irrespective of disease etiology, the long-term goal for treating ocular neovascular disease is to prevent neuronal loss.

## Materials and Methods

### Animals

Animals were obtained from Harlan Laboratories (Shardlow, UK), Charles River (Margate, UK) or in-house colonies and used according to UK Home Office (http://goo.gl/YLUFmF, last accessed 26th October 2015) and the Association for Research in Vision and Ophthalmology Statement for the Use of Animals in Ophthalmic and Vision Research guidelines (http://goo.gl/OwwfLZ, last accessed 26th October 2015). All animals received food and water *ad libitum*, in a 12-h day/night cycle, temperature-controlled environment.

When required, mice were anesthetized by intraperitoneal (IP) injection of xylazine (0.5 mg/kg) and ketamine (100 mg/kg) in water. Rats were anesthetized by IP injection of 37.5 mg/kg ketamine and 0.25 mg/kg medetomidine hydrochloride. Pupils were dilated with phenylephrine hydrochloride 2.5% and tropicamide 1% (Bausch and Lomb, Surrey, UK) before intravitreal injections.

### Ins2^Akita^

Ins2^Akita^ mice were obtained from Jackson Lab (Bar Harbor, ME, USA, stock number 003548). The Ins2^Akita^ mouse has a mutation in the insulin 2 gene, and develops hyperglycemia from approximately 3–4 weeks old.^8^ Experimental mice were generated by mating Ins2^Akita^ heterozygous males with C57BL/6 J females, and diabetes was confirmed by blood glucose measurements exceeding 250 mg/dl. Six-week-old heterozygous males of C57BL/6J background and age-matched non-diabetic siblings were used in the study. Mice were anesthetized and administered with 4 pmol in 1 *μ*l of either soluble VEGFR-2 (sVEGFR-2; 357-KD-050/CF; R&D systems, Abingdon, UK) or IgG_1_ control (110-HG-100; R&D Systems), via a bilateral intravitreal injection. Seven days later the intravitreal injections were repeated, and after a further 7 days the animals were killed by CO_2_ asphyxiation, the eyes removed and fixed in 4% paraformaldehyde (PFA) for TUNEL staining as described below. Animals used in this study were killed at 8 weeks old, and at death Ins2^Akita^ mice displayed significant hyperglycemia and reduced weight in comparison with C57Bl/6J controls (Table 1), in line with previous reports.^8^

### JR5558

JR5558 mice were discovered at The Jackson Laboratory, and bred and maintained from in-house colony as previously described.^9^ Both male and female age-matched littermates were used for experiments. Mice were anesthetized and administered with either 4pmol in 1 *μ*l of sVEGFR-2 or IgG_1_ control, via a bilateral intravitreal injection. After 7 days the animals were killed by CO_2_ asphyxiation, and the eyes enucleated and fixed in 4% PFA for TUNEL staining. Fluorescein angiography was carried out the day prior to intravitreal injection as well as tissue harvest, as detailed below.

### Fluorescein angiography

Fundus photography and retinal angiography were performed on conscious mice as previously described.^9^ Lesion number and area were quantified using ImageJ (http://imagej.nih.gov/ij/; provided in the public domain by the National Institutes of Health, Bethesda, MD, USA). Lesion area is represented in figures as the percentage of control; however, statistics were done on raw pixel measurements. Investigator (RF) was blinded to treatment groups.

### TUNEL staining

TUNEL staining was carried out on whole mount tissue. Retinas were dissected out and permeabilized in 3% Triton X-100 in PBS (T-PBS) for 2 h at room temperature (RT). The TUNEL protocol was performed according to the manufacturer's instructions (Promega, Southampton, UK), and the retinas were then stained with 1:500 biotinylated isolectin-B4 (IB4; Sigma-Aldrich, Dorset, UK) overnight at 4 °C, followed by 1:500 streptavidin labeled with AlexaFluor 594 for 2 h at RT. Tissue was washed in 0.3% T-PBS with 5 mM DAPI and flat mounted in Vectashield (Vector Laboratories, Peterborough, UK). To quantify TUNEL-positive neurons, we used a Zeiss 710 confocal microscope (Zeiss, Oberkochen, Germany), taking tile scans of the entire retina, with 30 *μ*m Z-stacks through the GCL and/or ONL using a × 10 magnification water-immersion objective. IB4 staining and morphological criteria discriminated non-neuronal (endothelial and glial) cells from neuronal cells. The total number of TUNEL positive cells in the GCL or ONL were counted, and investigator (RF) was blinded to treatment groups.

### Transport assay

Female Brown-Norway ex-breeder rats were used for all in vivo transportexperiments, due to ease and accuracy of intravitreal injection for quantification of axon transport. Rats were anesthetized and administered with either 20 pmol in 3 *μ*l of sVEGFR-2, or IgG_1_ control, via a bilateral intravitreal injection. After 7 days animals were injected with 2 *μ*l of a 0.75% solution of AlexaFluor 488-conjugated cholera toxin B subunit (CTB; Life Technologies, Paisley, UK) in sterile PBS. Following injection the needle was held in place for 1 min to prevent reflux of tracer. Rats were deeply anesthetized with pentobarbital sodium, before being transcardially perfuse-fixed with 75 ml of PBS, followed by 75 ml of 4% PFA, at 4, 8, 24 and 48 h post-CTB injection. Eyes, optic nerves and brains were removed for histology.

### Brain preparations

Brains were dehydrated (30% sucrose+0.1% sodium azide) and the SC sectioned (30 *μ*M) using a freezing microtome (SM2400; Leica Inc., Wetzlar, Germany). Orientation was confirmed through a needle mark to the right midbrain, visible on all sections.

Sections were blocked (0.3% triton, 5% normal goat serum (NGS) in PBS), counter-labeled with Nissl (1:200; Life Technologies) and DAPI (1:10 000; Life Technologies) and imaged using epifluorescent microscopy (DM6000 microscope; Leica Inc.). Images were taken using a 5 × air objective and merged together using Adobe Photoshop CS5 (Adobe Systems, San Jose, CA, USA) to represent the entire SC.

Approximately 10 sections per brain were chosen for analysis based on consistent central SC morphology and cerebral aqueduct shape and size. Quantitative analysis of CTB fluorescence intensity was measured using ImageJ software (http://imagej.nih.gov/ij/; provided in the public domain by the National Institutes of Health, Bethesda, MD, USA), and fluorescence measured in arbitrary units (0=black, 255=white).

Mean gray level fluorescence intensity was calculated by outlining the right and left superior colliculi with the same 'D'-shaped polygon (matching the shape of each colliculus). Intensity profiles for each treatment were created by measuring fluorescence from the outer edge of the SC towards the central midbrain at three evenly spaced regions. Mean data were plotted as a line graph of fluorescent intensity against distance and the area under the curve (AUC) calculated. AUC provided a measurement for cumulative fluorescence across the SC. Peak fluorescent intensity within these regions was also determined. Investigator (AO) was blinded to treatment groups.

### Optic nerve preparations

Optic nerves were dissected between the posterior globe and the optic chiasm, and post-fixed overnight in 4% PFA at 4 °C. The tissue was then washed three times in PBS, mounted in optimum cutting temperature compound (OCT) (TissueTek; Sakura Finetek, Thatcham, UK) and 10 *μ*m longitudinal frozen sections were prepared. The sections were mounted on microscope slides in Vectashield with DAPI. Tile scan images of the whole optic nerves were taken using a Zeiss 710 confocal microscope, with identical gain settings for each individual section. Mean fluorescence intensity was measured across the width of the optic nerve at 100 *μ*m intervals, using ImageJ software, plotted as a line graph against distance (along the nerve) and the AUC calculated using Prism software (GraphPad, La Jolla, CA, USA). Investigator (RF) was blinded to treatment groups.

### CTB uptake into the retina

Eyes were post-fixed overnight in 4% PFA following perfuse-fixation, at 4, 8, 24 and 48 h after intravitreal CTB injection. Retinas were dissected from the eyecups, and washed three times in PBS, before being whole mounted on glass slides in Vectashield with DAPI, with the GCL facing upwards. Tile scan images of the whole retinas were taken using a Zeiss 710 confocal microscope using a × 4 air objective, with identical gain settings for all tissue. Mean fluorescence intensity was measured across the retina using Adobe Photoshop, for all time points. Higher magnification images were captured using × 10 and × 40 water immersion objectives. Investigator (RF) was blinded to treatment groups.

### Quantification of retinal ganglion nuclei

Retinas of Brown-Norway rats from the anterograde transport experiments were permeabilized in 3% T-PBS for 2 h at RT, then incubated overnight with 1:500 biotinylated-IB4 at 4 °C, followed by 1:500 streptavidin labeled with AlexaFluor 594 for 2 h at RT. Tissue was washed in 0.3% T-PBS with 5 mM DAPI and flat mounted in Vectashield. To quantify RGCs, we used a Zeiss 710 confocal microscope (Zeiss, Oberkochen, Germany) with a × 10 water-immersion objective, to take 15 *μ*m Z-stacks through the GCL of each of the four retina petals. Maximum intensity projections were made of the images, and then processed using Adobe Photoshop to remove the vascular area from each picture. An area of approximately 0.5 mm^2^ at central, medial and peripheral regions of each petal (0.5, 2.0 and 3.5 mm from the optic nerve, respectively) was selected, and cells in these regions counted using ImageJ software (NIH), giving 12 images per whole mount and sampling approximately 3000 cells/retina. RGC counts were normalized to cells/mm^2^ before statistics were conducted. Investigator (RF) was blinded to treatment groups. Investigator (RF) was blinded to treatment groups.

### Phosphorylated p38 MAPK, Hsp27 and Tau immunostaining

Retinas of Brown-Norway rats from the CTB anterograde transport experiments were permeabilized in 3% T-PBS for 1 h, then blocked for 2 h in 5% NGS in 0.3% T-PBS at RT, before being incubated with antibodies for phosphorylated p38 MAPK (1:100; 9216 S; Cell Signaling Technology, Beverly, MA, USA), phosphorylated Hsp27 (1:100; 9709 S; Cell Signaling Technology), phosphorylated Tau (1:100; MN1020B; Life Technologies) or GFAP (1:200; Z0334; Dako, Cambridge, UK) overnight at 4 °C. Retinas were washed in 0.3% T-PBS, before being incubated with combinations of goat anti-mouse AlexaFluor 555, goat anti-rabbit AlexaFluor 555, goat anti-rabbit AlexaFluor 594 conjugated secondary antibodies (Life Technologies) or AlexaFluor 555-conjugated streptavidin (Life Technologies) for 2 h at RT. Tissue was washed in 0.3% T-PBS with 5 mM DAPI and flat mounted in Vectashield. A Zeiss 710 confocal microscope, with water-immersion × 10 and × 40 magnification objectives, was used to take 20 *μ*m Z-stacks through the GCL of IgG_1_ and sVEGFR-2 treated retinas, and maximum intensity projections of the cells were generated. Controls were no primary antibody and relevant IgG isotype controls. Investigator (RF) was blinded to treatment groups.

### Synapse protocol

Quantification of synapse number in the outer (OPL) and inner plexiform layers was quantified using immunohistochemistry, based on the method of Ippolito and Eroglu (2010)^15^ Briefly, 7-week-old C57Bl/6J mice (Charles River) were anesthetized and administered with either 4pmol in 1 *μ*l of sVEGFR-2 or IgG_1_ control, via a bilateral intravitreal injection. After 7 days the animals were deeply anesthetized with pentobarbital sodium, before being transcardially perfused with 10 ml of PBS. The eyes were enucleated and fixed in 4% PFA for 1 h. The lens was removed, and eyes were cryoprotected in 30% sucrose, embedded in OCT and sagittal cryostat sections (10 *μ*m) thaw-mounted onto glass slides. Sections were blocked in 20% NGS for 1 h at room temperature (RT), before rabbit anti-PSD-95 (1:200; Life Technologies) and mouse anti-synaptophysin (1:500; Abcam, Cambridge, UK) antibodies in 0.3% Triton X-100 in PBS (T-PBS) with 10% NGS were added overnight. Slides were then washed 3 x 10 min in PBS, before goat anti-mouse conjugated to AlexaFluor 488, and goat anti-rabbit conjugated to AlexaFluor 594 secondary antibodies in 0.3% Triton X-100 in PBS (T-PBS) with 10% NGS, were added for 2 h at room temperature. Slides were washed a further 4 × 10 min, before mounting in Vectashield with DAPI.

For quantification of synapse number, 5 *μ*m confocal scans were performed (optical section width, 0.38 *μ*m; 15 optical sections each), at the periphery, middle and optic nerve portions of each section, on 3 sections per eye (total 9 images). Maximum intensity projections for each image were generated, and synapse colocalization quantified as detailed previously.^15^ Investigator (RF) was blinded to treatment groups.

### Statistics

Statistical analyses were performed using GraphPad Prism software version 6 (GraphPad Software). For comparisons of two treatment groups, a Student's *t*-test was used. For analyzing three or more means, one-way analysis of variance (ANOVA) with a Newman–Keul's *post hoc* test was carried out. For multiple treatment groups over separate time points, a two-way ANOVA with a Newman–Keul's *post hoc* test was used. Results are means±S.E.M. unless stated, with each *N* representing an individual retina; N was at least three for each statistical analysis. *P*<0.05 was considered significant.

## Figures and Tables

**Table 1:** Weights and blood glucose of 8-week-old C57Bl/6 J and Ins2^Akita^ mice at the time of death

**Mouse strain**	***N***	**Weight (g)**	**Blood glucose (mg/dl)**
Wild type	10	24.7±2.3	218.3±30.4
Ins2^Akita^	9	21.5±1.6	509.0±77.8

## Abbreviations

AMD: age-related macular degeneration
ANOVA: analysis of variance
AUC: area under the curve
CATT: comparison of age-related macular degeneration treatments trials
CNV: choroidal neovascularization
CO_2_: carbon dioxide
CTB: cholera toxin B subunit
DAPI: 4',6-diamidino-2-phenylindole
GA: geographic atrophy
GCL: ganglion cell layer
GFAP: glial fibrilliary acidic protein
H_2_O_2_: hydrogen peroxide
Hsp27: heat-shock protein 27
IB4: isolectin-B4
IgG: immunoglobulin G
INL: inner nuclear layer
IOP: intraocular pressure
IP: intraperitoneal
NGS: normal goat serum
OCT: optimal cutting temperature compound
OHT: ocular hypertension
ONL: outer nuclear layer
OPL: outer plexiform layer
P38 MAPK: P38 mitogen-activated protein kinases
PBS: phosphate buffered saline
PFA: paraformaldehyde
Pi3K: phosphatidylinositide 3-kinase
PSD-95: postsynaptic density 95
RGC: retinal ganglion cell
RT: room temperature
SC: superior colliculus
S.E.M.: standard error of the mean
sVEGFR-2: soluble vascular endothelial growth factor receptor 2
TUNEL: terminal deoxynucleotidyl transferase dUTP nick end labeling
VEGF-A: vascular endothelial growth factor A
VEGFR-2: vascular endothelial growth factor receptor 2
