# Supplementary figures and images for: Distal retinal ganglion cell axon transport loss and activation of p38 MAPK stress pathway following VEGF-A antagonism
Source: Cell Death Dis. 2016 May 5;7(5):e2212–. doi: 10.1038/cddis.2016.110 (PMC4917649; doi:10.1038/cddis.2016.110)

A)

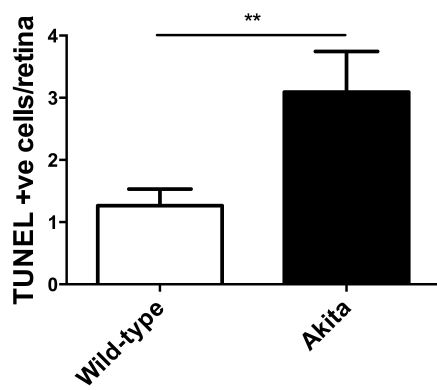

B)

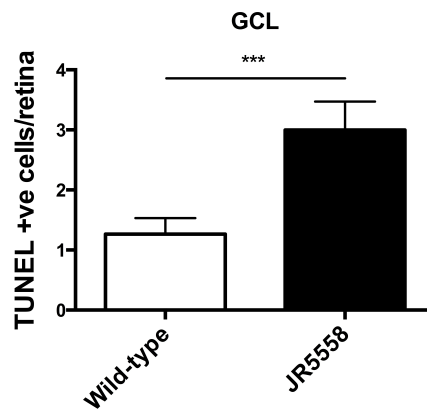

C)

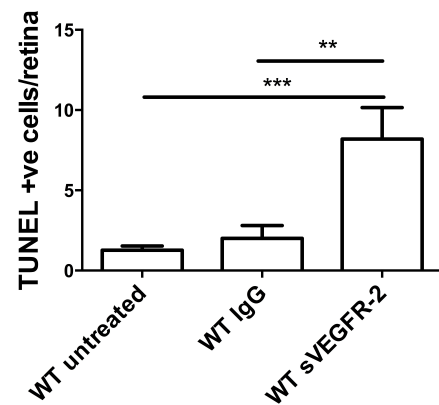

D)

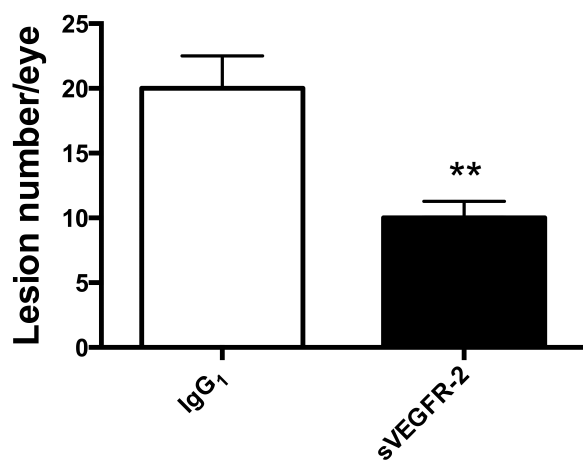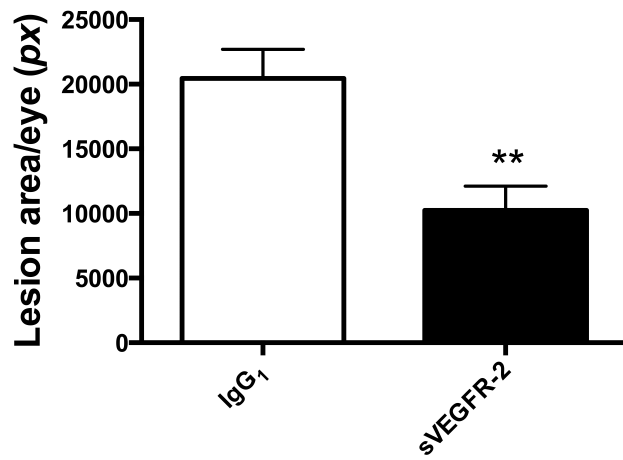

E)

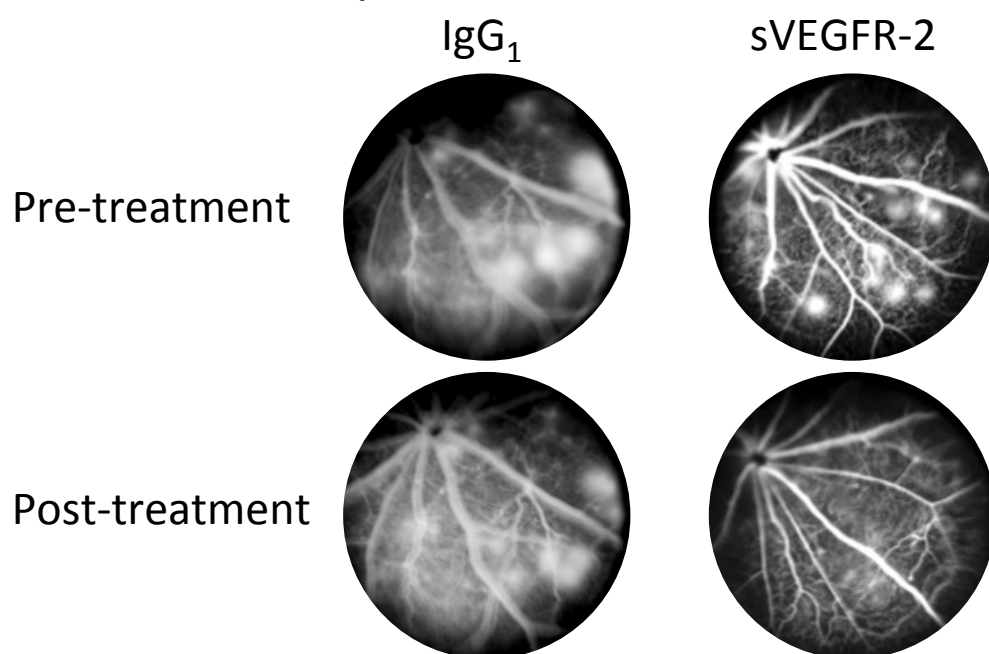

A)

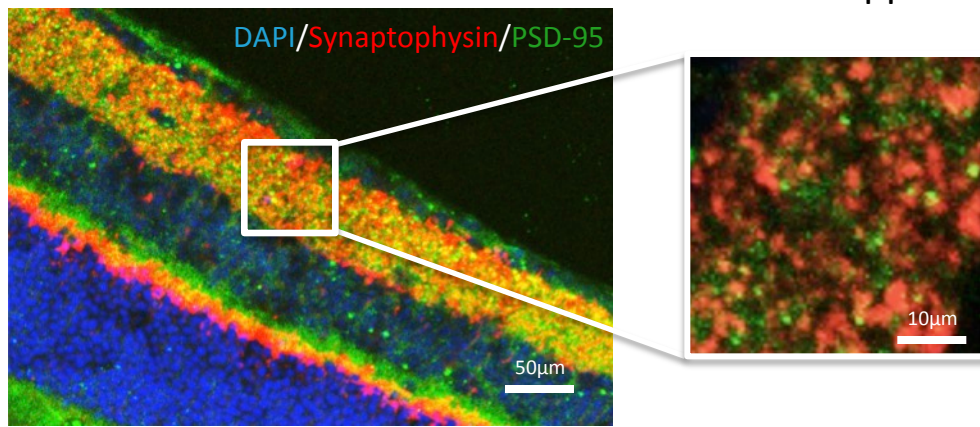

B)

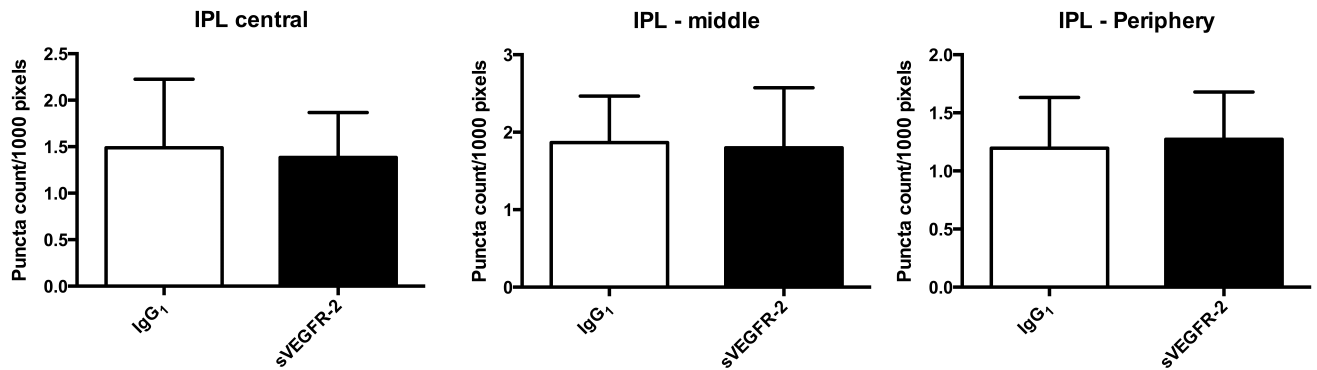

C)

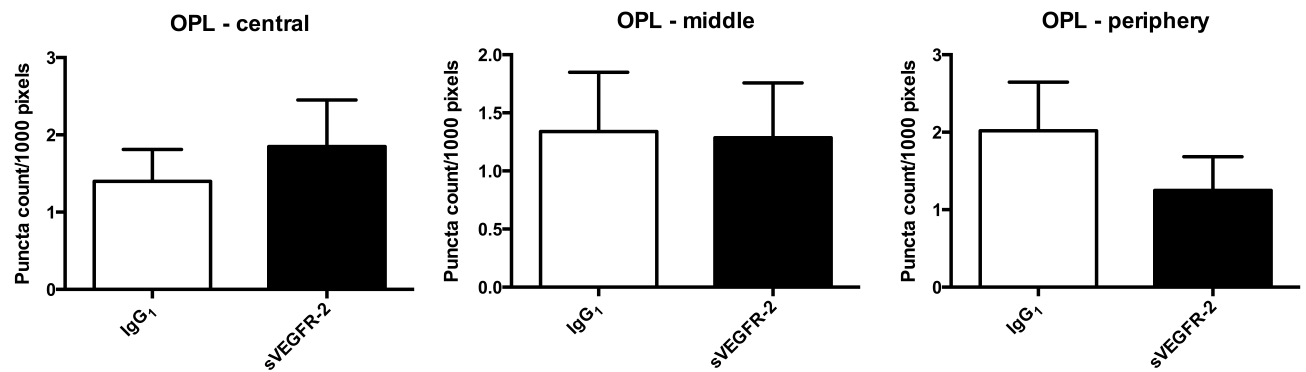

A)

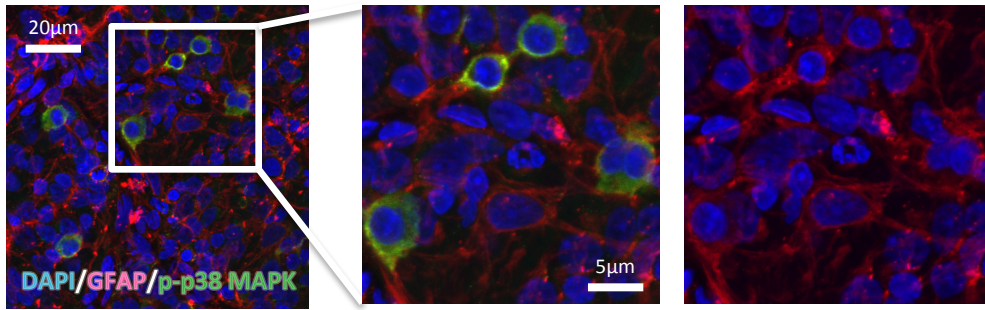

B)

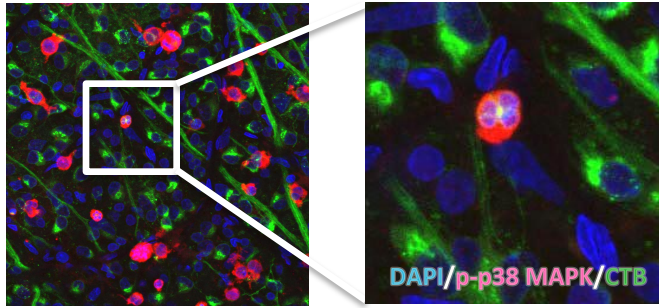

Supplement: Supplementary Figure 1 [file cddis2016110x1.pdf]
