# Supplementary material for: Distal retinal ganglion cell axon transport loss and activation of p38 MAPK stress pathway following VEGF-A antagonism
Source: Cell Death Dis. 2016 May 5;7(5):e2212–. doi: 10.1038/cddis.2016.110 (PMC4917649; doi:10.1038/cddis.2016.110)
Supplement: Supplementary Information [file cddis2016110x2.doc]

**Supplementary Figure 1.** Retinal neuronal cell death in Ins2^Akita^ and JR5558 mice, and response of JR5558 neovascular lesions to sVEGFR-2.

**A**) RGC death in untreated Ins2^Akita^ mice was significantly elevated above C57Bl/6J controls. The number of TUNEL-positive cells in the GCL was approximately 2.1-fold higher than wild-type. *N* = 10-15. **B**) Differences between RGC apoptosis in wild-type and JR5558 were also observed. TUNEL-positive cells in the GCL were found at levels 2.4-fold higher in JR5558 than C57Bl/6J. *N* = 10-15. **C**) Increases in apoptosis were additionally observed when wild-type mice were treated with sVEGFR-2. Cell death was increased 4.1-fold vs. IgG treatment, and 6.5-fold vs. untreated control. *N* = 8-15. **D**) Neovascular lesions in JR5558 spontaneous CNV mice were affected by VEGF-A neutralization. Both lesion number (**left panel**) and lesion area per eye (**right panel**) were significantly reduced by sVEGFR-2 in comparison to IgG_1_ control. *N* = 5-6. **E**) Representative fluorescein angiograms of IgG_1_ (**left images**) and sVEGFR-2 (**right images**) treated eyes, at pre-treatment (**top images**) and post-treatment (**bottom images**) time points. Original magnification = x4. ** *P* < 0.01, *** *P* < 0.001. Data are given as means ± SEM.

**Supplementary Figure 2.** VEGF-A antagonism does not affect synaptic architecture in the adult mouse retina. C57Bl/6J mice were injected intravitreally with IgG_1_ or sVEGFR-2, then 7 days later the tissue was harvested, and frozen sections prepared. **A**) Sections were stained with synaptophysin (pre-synaptic marker; red), PSD-95 (post-synaptic marker; green) and DAPI (blue). **Left panel** shows low magnification (x10) images of staining. Quantification of the number of co-localised synaptophysin and PSD-95 puncta was carried out using high magnification (x63; **right panel**). Images were taken at the periphery, middle, and optic nerve portions of each section, on 3 sections per eye (total 9 images). No significant differences in puncta number were observed between IgG_1_ and sVEGFR-2 treatments in the IPL (**B**) or OPL (**C**), in central (**left graphs**), middle (**middle graphs**) or peripheral (**right graphs**) regions of the retina. *N* = 6. Data are given as means ± SEM.

**Supplementary Figure 3.** Phosphorylated p38 MAPK expression is elevated predominantly in astrocytes, but also RGCs following VEGF neutralization. **A**) Phosphorylated p38 MAPK (green; p-p38 MAPK) colocalises with GFAP staining (red), indicating mainly astrocyte expression. Blue = DAPI. **Left panel** = x10, **middle panel** = x40 magnification, **right panel** = x40 minus p38 MAPK. **B**) However, some CTB (green) positive cells, also express p-p38 MAPK. **Left panel** = x10, **middle panel** = x40 magnification.
